# A T-type channel-calmodulin complex triggers αCaMKII activation

**DOI:** 10.1186/s13041-017-0317-8

**Published:** 2017-08-11

**Authors:** Hadhimulya Asmara, Ileana Micu, Arsalan P. Rizwan, Giriraj Sahu, Brett A. Simms, Fang-Xiong Zhang, Jordan D. T. Engbers, Peter K. Stys, Gerald W. Zamponi, Ray W. Turner

**Affiliations:** 1 Department of Cell Biology and Anatomy, University of Calgary, Calgary, AB T2N 4N1 Canada; 2 Department of Clinical Neurosciences, University of Calgary, Calgary, AB T2N 4N1 Canada; 3 Department of Physiology and Pharmacology, University of Calgary, Calgary, AB T2N 4N1 Canada; 4 Hotchkiss Brain Institute, University of Calgary, Calgary, AB T2N 4N1 Canada; 5 Alberta Children’s Hospital Research Institute, Cumming School of Medicine, University of Calgary, Calgary, AB T2N 4N1 Canada; 6 HRIC 1AA14, University of Calgary, 3330 Hospital Dr. N.W, Calgary, AB T2N 4N1 Canada

**Keywords:** Calmodulin, Cav3.1, T-type channel, αCaMKII, Hippocampus, Purkinje cell, Cerebellum

## Abstract

Calmodulin (CaM) is an important signaling molecule that regulates a vast array of cellular functions by activating second messengers involved in cell function and plasticity. Low voltage-activated calcium channels of the Cav3 family have the important role of mediating low threshold calcium influx, but were not believed to interact with CaM. We find a constitutive association between CaM and the Cav3.1 channel at rest that is lost through an activity-dependent and Cav3.1 calcium-dependent CaM dissociation. Moreover, Cav3 calcium influx is sufficient to activate αCaMKII in the cytoplasm in a manner that depends on an intact Cav3.1 C-terminus needed to support the CaM interaction. Our findings thus establish that T-type channel calcium influx invokes a novel dynamic interaction between CaM and Cav3.1 channels to trigger a signaling cascade that leads to αCaMKII activation.

**Electronic supplementary material:**

The online version of this article (doi:10.1186/s13041-017-0317-8) contains supplementary material, which is available to authorized users.

## Introduction

Calcium channels mediate a wide range of cellular functions that involve activation of calcium-dependent enzymes that can shape neuronal output [1]. Members of the Cav3 calcium channel family are designed to regulate calcium entry in the subthreshold voltage range to control action potential generation [2–4] and transmitter release [5–8]. Dysregulated Cav3 channels contribute to pathologies such as epilepsy, chronic pain, and ataxia [1, 9–11]. It has been well established that high voltage-activated (HVA) calcium channels interact with the calcium sensing protein calmodulin (CaM) to regulate channel function [12–14]. Moreover, calcium entry through HVA calcium channels can trigger a second messenger cascade by which CaM activates Ca^2+^/CaM-dependent protein kinase II (CaMKII) that can modify cell activity and long-term plasticity [15–19]. In contrast, the Cav3 channel family is reported to lack CaM binding consensus motifs that would permit calcium-dependent interactions with CaM [20, 21] and there are no reports of Cav3 calcium influx triggering a signaling cascade for CaMKII activation. The available data then suggest that activation of CaM-CaMKII signaling by voltage-dependent calcium channels is restricted to HVA calcium channels that open in response to large membrane depolarizations.

We now report that under resting conditions, CaM constitutively associates with the C-terminus of Cav3.1, as revealed in both a heterologous expression system and rodent brain. In response to Cav3.1-mediated calcium influx the Cav3.1-CaM association is lost, accompanied by phosphorylation of αCaMKII in both a heterologous expression system and neurons in hippocampus and cerebellum. These results are important in providing the first evidence of a role for Cav3.1 channels in triggering αCaMKII as a second messenger involved in mediating a host of cellular functions.

## Methods

### Animal care and tissue dissection

Sprague-Dawley rats and C57/BL/6 mice were obtained from Charles River and maintained according to guidelines of the Canadian Council for Animal Care, and euthanized according to Standard Operating Procedures defined by the University of Calgary Animal Resource Center. All chemicals were obtained from Sigma (Oakville, ON, Canada) unless otherwise indicated. Animals at P19–40 were anaesthetized by isofluorane inhalation until unresponsive to ear pinch and the brain dissected out in the presence of ice cold sucrose-cutting solution composed of (in mM): 215 sucrose, 25 NaHCO_3_, 20 D-glucose, 2.5 KCl, 1.25 NaH_2_PO_4_ and 3 MgCl_2_ preoxygenated by carbogen (95% O_2_, 5% CO_2_) gas [22]. For coimmunoprecipitations brain tissue was transferred to a lysis buffer medium comprised of (in mM): 150 NaCl, 50 Tris pH 7.5, 0.5% sodium-decholic acid, 1% NP-40, 2.5 EGTA, 0–3 CaCl_2,_ and 1 tablet complete protease inhibitor cocktail (EDTA free) in 10 ml, and stored at 4 °C. A separate set of experiments prepared brain tissue to create homogenates in different buffered levels of calcium concentration according to Lai et al. [23]. For tissue slice histochemistry sagittal cerebellar tissue slices of 150 μm thickness were prepared from P30-P40 male rats by Vibratome in ice-cold sucrose-cutting solution as above. For tissue destined for recordings, slices were incubated at 37 °C in artificial cerebrospinal fluid (aCSF) composed of (mM): 125 NaCl, 1.0 KCl, 1.5 CaCl_2_, 1.5 MgCl_2_, 25 NaHCO_3_, and 25 D-glucose bubbled with carbogen gas, with a subset exposed to aCSF containing 50 mM [K]o for 10 min prior to immediate removal and fixation for immunolabel processing. Calcium influx was restricted to Cav3 calcium channels throughout by cutting tissue slices and maintaining tissue in aCSF containing 30 μM Cd^2+^, 1 μM TTX, 10 μM DNQX and 25 μM or 100 μM DL-AP5.

Low-density cultures of hippocampal neurons were prepared from P0-P1 pups of C57/BL/6 mice initially anesthetized by placing on ice for 5–10 min. Dissected hippocampi were dissociated by papain treatment and trituration and plated on poly-l-lysine and laminin-coated glass coverslips in 24 well culture dishes at a density of 3500–5300 cells/cm^2^. After plating, cells were allowed to grow on coverslips for 10 days in medium composed of (in mM): free neurobasal medium (BME), 1% B-27 supplement, 5% FBS, 0.6% glucose, 1 Na-Pyruvate, 2 L-glutamine, 10 HEPES, 1% Pen-strep. All cell culture chemicals were obtained from Life Technologies (Burlington, ON, Canada).

### Molecular biology

Wild-type human Cav3.1b cDNA was donated by T. Snutch (Vancouver, BC, Canada) (GenBank accession number AF134986.1), HA-Cav3.1 and Kir2.1 cDNA from E. Bourinet (Institut de Génomique Fonctionnelle, Montpellier, FR), and CaM cDNAs by J. Adelman (Vollum Institute, OR, USA). GFP-Cav3.1 and GFP-αCaMKII were subcloned into a GFP mammalian vector (pCMV6-AN-GFP) (OriGene Technologies, Rockville, MD, USA) and mKate-CaM prepared from a pmKate2-N mammalian expression vector (Axxora, Farmingdale, NY, USA). CaMKIIN was amplified from a mouse hippocampal cDNA library (RIKEN) and cloned into the pcDNA3.1 vector (Addgene, Cambridge, MA, USA) [24]. To create a deletion of the C-terminus, PCR with specific primers was performed on Cav3.1 outside of the C-terminus region, followed by sequencing and subcloning into the pCMV6-AN-GFP vector (OriGene Technologies, Rockville, MD, USA) or the pcDNA3.1 vector (Addgene, Cambridge, MA, USA) and sequenced for final confirmation. The GFP and mKate tags were attached to the N-terminal regions of target proteins. To create the Cav3.1 pore mutant, a single point mutation PCR was performed on E354K in the pore region with the primer sequences: 5′-ACAGGTCGTTGAGCCGC-3′, followed by sequencing and subcloning into the pcDNA3.1 vector (Addgene, Cambridge, MA, USA) and sequenced for final confirmation.

### Transient transfection

cDNA constructs were transiently transfected into tsA-201 cells using the calcium phosphate method [25]. Coimmunoprecipitation experiments used 5 μg cDNA for each of Cav3.1, HA-Cav3.1, CaM, CaM1234, and GFP-Cav3.1 lacking the C-terminus (GFP-Cav3.1ΔCT). FRET studies used 1 μg cDNA of GFP-Cav3.1 and mKate-CaM, and live cell imaging experiments and immunocytochemistry used 1 μg Cav3.1 or Cav3.1ΔCT or Cav3.1 pore mutant, 1 μg CaM, and 0.7 μg GFP-αCaMKII. tsA-201 were also transfected with 0.3 μg Kir2.1 cDNA where indicated. Transfected cells were incubated at 37 °C (5% CO_2_) for 24 h and then transferred to a 30 °C incubator (5% CO_2_) for 48–72 h prior to imaging studies or incubated at 30 °C for 48 h before being lysed using a buffered calcium concentration buffer for coimmunoprecipitations.

### Coimmunoprecipitation assays

tsA-201 cells transiently transfected with Cav3.1 or Cav3.1ΔCT and either CaM or CaM1234 were lysed in a buffer containing (in mM): 150 NaCl, 50 Tris, 2.5 EGTA, 0.5% sodium-decholic acid, 1% NP-40, 0–3 CaCl_2_, pH 7.5. The nominal level of free calcium in solutions was calculated using Maxchelator software (http://maxchelator.stanford.edu/CaEGTA-NIST.htm) to conduct coimmunoprecipitations in the presence of different levels of free calcium concentration ranging from 0 to 1 mM (see [23]). Brain tissue was prepared in cell lysis buffer using a hand held glass homogenizer. Lysates from tsA-201 cells were centrifuged at 13,000 rpm for 1 min at 4 °C and supernatants were transferred to new tubes. Solubilized proteins were incubated with 1 μg of rabbit anti-Cav3.1 antibody (1:1000) corresponding to amino acid residues of the I-II linker of rat Ca_V_3.1 [22], or with anti-CaM antibody (1:1000; Millipore, Etobicoke, ON, Canada) and 30 μl of Protein G beads (Life Technologies, Burlington, ON, Canada) while rotating overnight at 4 °C. Coimmunoprecipitates were washed three times with phosphate buffered saline (PBS) in (mM): 137 NaCl, 2.7 KCl, 1 Na_2_HPO_4_, 1.8 KH_2_PO_4_, pH 7.4 reconstituted in an equal volume of 2X loading buffer (in mM): 100 Tris, 100 2-mercaptoethanol, 4% SDS, 0.02% bromophenol blue, 20% glycerol, pH 6.8 and incubated at 100 °C for 5 min. Eluted samples were loaded on 6–10% Tris-glycine gel and resolved using SDS-PAGE. Samples were transferred to 0.2 μm PVDF membrane (Millipore, Etobicoke, ON, Canada) and Western blot analysis performed using a mouse monoclonal anti-CaM antibody (1:1000; Millipore, Etobicoke, ON, Canada) or a mouse monoclonal anti-Hemagglutinin (HA) antibody (0.4 μg; Roche, Mississauga, ON, Canada). Secondary antibodies were conjugated to horseradish peroxidase (HRP; 1:5000; Molecular Probes, Eugene, OR, USA) and reacted with ECL solution (Life Technologies, Burlington, ON, Canada).

### GST-pull down assay

To prepare the GST-C-terminus of Cav3.1, we transformed Cav3.1 C-terminus GST fusion protein plasmids into BL21 and induced the expression of recombinant proteins by 0.5 mM isopropyl-β-D-1-thiogalactopyranoside. GST fusion proteins were purified from bacteria using glutathione-Sepharose 4B beads (Life Technologies, Burlington, ON, Canada) according to the protocol recommended by the manufacturer. The GST tag was then cleaved using Precision Protease (Life Technologies, Burlington, ON, Canada). Complete cleavage of the fusion protein was verified using Western blotting. The recombinant Cav3.1 C-terminus peptides were then applied to CaM sepharose beads and incubated overnight. The CaM sepharose beads were washed three times with PBS, reconstituted in equal volume of loading buffer (100 mM Tris, 4% SDS, 0.02% bromophenol blue, 20% glycerol, 100 mM 2-mercaptoethanol, pH 6.8) and incubated at 100 °C for 5 min. Eluted samples were loaded on a 6–10% Tris-glycine gel and resolved using SDS-PAGE. Samples were transferred to 0.2 μm PDVF membrane (Millipore, Etobicoke, ON, Canada) and western blot analysis was performed using a goat anti-Cav3.1 antibody targeted to the C-terminus region of the channel (1:1000; Santa Cruz, Dallas, TX, USA). The secondary antibody used was a goat antibody conjugated to HRP (1:5000; Molecular Probes, Eugene, OR, USA) and reacted with ECL solution (Life Technologies, Burlington, ON, Canada).

### Western blot

Cerebellar tissue slices lysates of 150 μm thickness were incubated for 10 min in any of three conditions: low [K]o (1 mM), high [K]o (50 mM), or high [K]o with mibefradil (1 μM) and Ni^2+^ (300 μM). Homogenates were then made in the same solutions using a hand held glass homogenizer. Eluted lysates were loaded on 6–10% Tris-glycine gel and resolved using SDS-PAGE. Samples were transferred to 0.2 μm PVDF membrane (Millipore, Etobicoke, ON, Canada) and Western blot analysis performed using a monoclonal mouse anti-αCAMKII (1:1000; Santa Cruz, Dallas, TX, USA) or a polyclonal rabbit anti-p-αCAMKII (Thr286) (1:1000; Santa Cruz, Dallas, TX, USA). The secondary antibodies used were the appropriate mouse or rabbit antibodies conjugated to HRP (1:5000; Molecular Probes, Eugene, OR, USA) and reacted with ECL solution (Life Technologies, Burlington, ON, Canada).

### Live cell fluorescence spectral confocal imaging

Cultured tsA-201 cells were transiently transfected with GFP-Cav3.1 and mKate-CaM constructs for FRET imaging. Cells were seeded onto poly-L-lysine coated 35 mm plates. Cells were then incubated for 24 h at 37 °C and 5% CO_2_, washed and replaced with colorless imaging medium (in mM): 148 NaCl, 3 KCl, 10 HEPES, 0 or 3 CaCl_2_, 8 glucose, 1 MgCl_2,_ pH 7.3 at 25 °C. Cultured cells were examined with a Nikon Eclipse C1si spectral confocal laser-scanning microscope with a 40×/1.3NA oil immersion objective. For FRET imaging GFP-Cav3.1 was excited at 457 nm and mKate-CaM at 561 nm. Emission spectra of GFP and mKate were recorded between 400 nm to 750 nm. The FRET signal was measured every 10 s for 450 s.

To separate the fluorescence signals of GFP and mKate spectral images were linearly unmixed using ImageTrak software (P.K. Stys, http://www.ucalgary.ca/styslab/imagetrak) [26], collapsing a 32 channel spectral image into a two channel image representing the integrated intensities of GFP and mKATE fluorescence emissions. The FRET response was quantified as follows: at each time point, the mean pixel intensity of each fluorophore from the linearly unmixed image within specific cellular ROIs representing the cell cytoplasm was first calculated. The mean mKate intensity was divided by the mean GFP intensity to yield a ratio. Changes over time were expressed as a % change vs. this ratio at time 0. Expressed in mathematical terms:

$$ \Delta \mathrm{R}\left(\mathrm{t}\right)=\frac{{\mathrm{F}}_{\mathrm{mKate}}\left(\mathrm{t}\right)}{{\mathrm{F}}_{\mathrm{GFP}}\left(\mathrm{t}\right)}\hbox{-} \frac{{\mathrm{F}}_{\mathrm{mKate}}(0)}{{\mathrm{F}}_{\mathrm{GFP}}(0)}\times 100\% $$

Where:

- ΔR(t) is the % change of mKate:GFP intensity ratio as a function of time (positive values mean an increase in mKate emission relative to GFP, implying greater FRET and therefore closer physical proximity of the two fluorescent proteins).
- F_mKate_(t) and F_GFP_(t) are the mean fluorescence intensities of the two fluorophores as a function of time
- F_mKate_(0) and F_GFP_(0) are the mean fluorescence intensities of the two fluorophores at time 0

For tests using GFP-αCaMKII expression tsA-201 cells were placed in colorless imaging medium comprised of (in mM): 148 NaCl, 1 or 50 KCl, 10 HEPES, 3 CaCl_2_, 8 Glucose, 1 MgCl_2_, pH 7.4 at 25 °C. For tests using GFP-αCaMKII transfection of hippocampal cultures, cells were transferred to aCSF containing 30 μM Cd^2+^, 1 μM TTX, 10 μM DNQX and 25 μM or 100 μM DL-AP5. The GFP-αCaMKII was excited at 457 nm and fluorescence signal in the cytoplasmic and nuclear compartment of cells was measured (ImageTrak). Images were recorded every 5 or 10 s for 250 or 400 s in control (1 mM [K]o) and test media (50 mM [K]o), and in the case of Fig. 4a, b, returned to aCSF containing low [K]o. The aggregation was calculated as the change in pixel variance of GFP fluorescence (ΔSD) presented as a percentage change from control variance (SD0G) to quantify the degree of GFP-αCaMKII aggregation (ΔSD/SD0G (%)) (Figs. 4 and 6; Additional file 5: Figure S5). Alternatively, GFP-αCaMKII aggregation was calculated as the mean fluorescence intensity change (ΔF) presented as a change from control mean (F0) (Figs. 5 and 7; Additional file 8: Figure S8).

### Immunohistochemistry

Tests for phosphorylation of GFP-αCaMKII and phosphorylation of αCaMKII were conducted on cultured tsA-201 cells transiently transfected with cDNA, mouse hippocampal cell cultures, and rat cerebellar tissue slices maintained as above and exposed to control or test conditions (1 mM or 50 mM [K]o). Cultured cells and tissues were then quickly washed in PBS and fixed by exposure to 4% paraformaldehyde (pH 7.4) at room temperature for 1 h and overnight at 4 °C. The cells or tissue were transferred to a working solution of 3% normal donkey or horse serum (Jackson Immuno-Research, West Grove, PA, USA), 0.2% TWEEN and 2% dimethylsulphoxide in phosphate buffer (PB). Primary antibodies were reacted for 48 h at 4 °C and washed in working solution 3 X 15 min and secondary antibodies for 4 h at room temperature. Primary antibodies corresponded to a monoclonal mouse anti-p-αCAMKII (Thr286) (1:500; Life Technologies, Burlington, ON, Canada). Secondary antibodies (1:1000) were the appropriate AlexaFluor-488 or −594 conjugated rabbit IgGs (Molecular Probes, Eugene, OR, USA). After washing in PB, sections were mounted on gel-coated slides, coverslipped with anti-fade medium and stored at −20 °C. Controls consisted of omitting the primary antibodies.

### Modeling

A single compartment model of Cav3 T-type calcium channels was modified from data originally presented in Engbers et al. [27]. The model had three conductances including Cav3, HCN and a leak current. Cav3 current was based on recordings from rat cerebellar Purkinje cells, and assigned the parameters of Va −30 mV, ka −4.6, λ_act_ 3 msec; V_h_: −68 mV, k_h_: 6.8, λ_inact_ 12 msec; E_Ca_ = 40 mV; single channel conductance 9 pS. Parameters for I_H_ were Vh −80 mV, k 3, λ_act_ 200 ms; E_H_ = −20 mV. E_leak_ = −77 mV. Calcium diffusion was modeled using 10 hemispherical compartments with radii of 20–200 nm (20 nm increments) using a calcium diffusion coefficient of 220 μm^2^/msec. Simulations were run with custom made scripts in Matlab r2016a [27].

### Statistical analysis

Statistical analysis was performed using Igor software (Wavemetrics, Lake Oswego, OR, USA) or IBM SPSS Statistics 20 Software (Armonk, NY, USA). The homogeneity of variances was assessed with Levene’s and Bartlett’s tests. Statistical significance was determined using a paired or unpaired Student’s *t*-test, one-way analysis of variance (ANOVA), or with the non-parametric multiple comparison Dunn-Holland-Wolfe and Mann-Whitney Wilcoxon tests as appropriate. Statistical significance was set at *p* < 0.05 with * *p* < 0.05, ** *p* < 0.01, ns, not significant. The average values are presented either as mean ± SEM or SD as indicated.

## Results

### Cav3.1 coimmunoprecipitates with CaM in a calcium-dependent manner

**Fig. 1:**
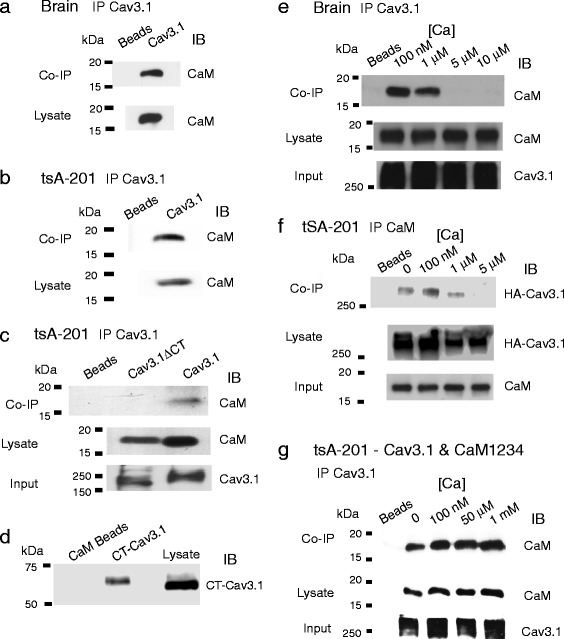
Cav3.1 channels exhibit a calcium-dependent association with CaM. Biochemical analysis of binding interactions between Cav3.1 and CaM using the indicated antibodies to immunoprecipitate (IP) or immunoblot (IB). The experiments are representative of at least 3 repetitions. Input lanes reflect controls to verify the efficiency of the IP step should IB antibodies fail to reveal co-IPs. **a**, **b** Cav3.1 channels co-IP with CaM from rat brain lysate (**a**) and homogenates of tsA-201 cells coexpressing Cav3.1 and CaM (**b)**. Co-IPs were conducted in the presence of 100 nM calcium. **c** The Cav3.1-CaM co-IP is lost for a construct lacking the Cav3.1 C-terminus (Cav3.1ΔCT). Co-IPs were conducted in the presence of 100 nM calcium. **d** A GST-pull down experiment between the C-terminus of Cav3.1 (CT-Cav3.1) onto CaM beads. GST-CT-Cav3.1 was grown in bacteria, and the GST tag subsequently cleaved off as the GST non specifically bound to CaM beads. CaM beads were instead incubated with purified recombinant Cav3.1 C-terminal peptides in the presence of 100 nM calcium and the sample eluted and run on a Western blot. The blot was probed with an antibody targeting the Cav3.1 C-terminus. **e**, **f** Co-IP tests conducted in the indicated calcium concentrations reveal that Cav3.1 associates with CaM only below 5 μM calcium. Co-IPs were conducted from rat brain lysates (**e**) or from homogenates of tsA-201 cell expressing HA-Cav3.1 and CaM (**f**). In (**f**) an HA antibody was used to immunoblot the HA-Cav3.1 conjugate. **g** Co-IPs from lysates of tsA-201 cells coexpressing Cav3.1 and the CaM EF-hand mutant CaM1234 reveals that CaM1234 and Cav3.1 interact in the presence of 0–1 mM calcium

We tested the potential for Cav3.1 and CaM to physically associate using coimmunoprecipitation, first performed in the presence of a buffered calcium concentration of 100 nM to simulate resting conditions of intracellular calcium (see Methods). All results for coimmunoprecipitations reflect data from 3 separate experiments. We found that Cav3.1 coimmunoprecipitated with CaM from rat brain lysate (Fig. 1a), indicating that CaM and Cav3.1 channels are part of a protein complex. To better define the underlying molecular determinants, we coexpressed cDNAs for CaM and Cav3.1 channels in tsA-201 cells and again were able to coimmunoprecipitate Cav3.1 and CaM (Fig. 1b). The interaction of CaM with Cav1.2 (L-type) calcium channels is known to involve the C-terminus [12]. To determine the potential involvement of the Cav3.1 C-terminus we coexpressed CaM and a Cav3.1 mutant construct lacking the C-terminus (Cav3.1ΔCT), and found no coimmunoprecipitation (Fig. 1c), indicating that the C-terminus is an essential element in a Cav3.1-CaM channel complex. To better define if the association between Cav3.1 and CaM was direct or indirect we used a binding assay involving purified CaM and internal segments of the Cav3.1 channel. This analysis revealed interactions between CaM and the Cav3.1 C-terminus, a result consistent with a direct protein-protein interaction (Fig. 1d).

It is known that at resting levels of calcium Cav1.2 channels are preassociated with ApoCaM and that an increase in the internal concentration of calcium leads to an increase in CaM binding affinity for the channel and a structural rearrangement of the CaM channel complex [13, 28–30]. To test the relative calcium dependence of the association between Cav3.1 and CaM, we immunoprecipitated Cav3.1 in the presence of specific levels of buffered calcium [23]. In this way we could test for potential constitutive binding of CaM in nominally zero calcium, near resting levels of calcium in neurons (100 nM), and in progressively higher but physiological levels of calcium [31]. CaM coimmunoprecipitated with Cav3.1 at 0 and 100 nM levels of calcium from lysates of either rat brain or tsA-201 cells expressing Cav3.1 and CaM (Fig. 1e, f) (Additional file 1: Figure S1). However, the Cav3.1-CaM coimmunoprecipitation was lost in the presence of 5 μM or greater calcium concentrations in both rat brain and tsA-201 cells (Fig. 1e, f; Additional file 1: Figure S1). To test if the loss of a Cav3.1-CaM association reflects a calcium-dependent interaction with CaM we coexpressed in tsA-201 cells Cav3.1 and the calcium binding deficient CaM mutant CaM1234, and found that CaM1234 coimmunoprecipitated with Cav3.1 for all concentrations of calcium tested (Fig. 1g).

These results indicate that the Cav3.1-CaM association occurs preferentially in low or resting levels of calcium, suggesting that Cav3.1 channels are constitutively associated with CaM. In contrast, there is a calcium-dependent dissociation of CaM from the Cav3.1 channel, revealing a process that is distinctly different from that reported between HVA calcium channels and CaM.

### Dynamics of Cav3.1 and CaM interactions revealed by FRET

**Fig. 2:**
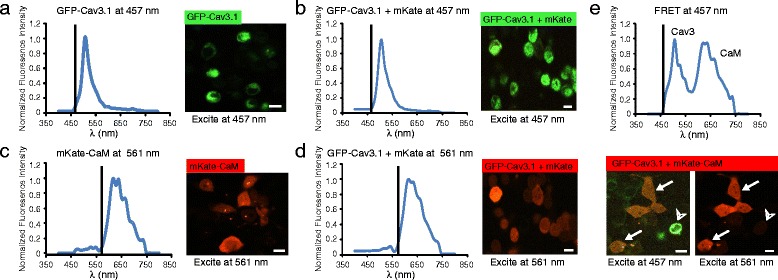
Cav3.1 and CaM associate at a level that supports FRET. Fluorescence spectral confocal images of tsA-201 cells expressing constructs of GFP-Cav3.1 (donor molecule) or mKate-CaM (acceptor molecule) excited at either 457 nm or 561 nm. Plots show the excitation (*vertical line*) and the associated emission spectrum for each condition. **a**, **b** Applying excitation at 457 nm to cells expressing either GFP-Cav3.1 alone (**a**) or in conjunction with the mKate acceptor molecule (**b**) results in a typical GFP emission spectrum. **c**, **d** Applying excitation at 561 nm to cells expressing either mKate-CaM alone (**c**) or in conjunction with GFP-Cav3.1 (**d**) results in a pure mKate emission spectrum. **e** Coexpressing GFP-Cav3.1 and mKate-CaM results in a double emission profile when excitation at 457 nm promotes FRET in a subset of cells (*arrows*). A control image of the same cells excited by a 561 nm laser line at right reveals that a GFP-Cav3.1 expressing cell (*arrowhead*) that does not exhibit FRET failed to express the mKate-CaM construct. All results were derived from at least 3 separate experiments. Scale bars 10 μm

To test the dynamics of the association between Cav3.1 and CaM in an intact cellular environment, we conducted FRET experiments in tsA-201 cells. For this we created constructs of a Cav3.1 channel with GFP attached to the N-terminus as a donor fluorophore (GFP-Cav3.1) and CaM with the red fluorophore mKate attached to its N-terminus (mKate-CaM) as an acceptor molecule. All tests on FRET were repeated in 3-5 cell culture plates to detect and assess emission from a large set of Regions of Interest (ROI). Control tests established that excitation using a 457 nm confocal laser line produced an emission profile characteristic of GFP in cells expressing only GFP-Cav3.1 (Fig. 2a), or cells expressing GFP-Cav3.1 together with a cDNA encoding the mKate molecule alone (Fig. 2b). By comparison, excitation at 561 nm resulted in an emission profile for mKate in cells transfected with mKate-CaM (Fig. 2c) as well as in cells expressing the mKate molecule and GFP-Cav3.1 (Fig. 2d). Importantly, coexpressing cDNA for both GFP-Cav3.1 and mKate-CaM allowed a single excitation line at 457 nm to evoke a double peaked emission spectrum indicative of FRET between GFP and mKate (Fig. 2e). We could also detect single cells that exhibited only green fluorescence when excited at 457 nm and no signal upon excitation at 561 nm, revealing that FRET was only observed in cells that expressed both cDNAs (Fig. 2e). These results are important in revealing that the association between Cav3.1 and CaM under resting conditions occurs at distances that are sufficiently close to support FRET.

Given that coimmunoprecipitation experiments indicated a calcium-dependent loss of the Cav3.1-CaM band on western blots (Fig. 1e, f), we used FRET to further test the calcium dependence of the Cav3.1-CaM association in live cells (measured in all cases from defined ROIs pooled from 3 to 6 separate experiments). Under normal conditions, tsA-201 cells display a depolarized membrane potential that leads to a voltage-dependent inactivation of Cav3.1 calcium channels. To allow these cells to rest at a more hyperpolarized potential we coexpressed cDNA for the inward rectifying potassium channel Kir2.1 [32]. Coexpressing Kir2.1 cDNA (0.3 μg) with GFP-Cav3.1 and mKate-CaM promoted a resting potential of −57.2 mV ± 2.8 mV in 1 mM [K]o, and a depolarization to −24.1 mV ± 5.8 mV (*n* = 3) in the presence of 50 mM [K]o. Modeling studies of the degree of calcium accumulation near the mouth of a Cav3 calcium channel pore indicate peak values as high as 50 μM [33]. We repeated these studies for Cav3 current parameters recorded from cerebellar Purkinje cells and previously modeled with respect to activation of BK potassium channels [27]. Here the model reports an increase in predicted [Ca]i to >35 μM at 20 nm distance, and 7 μM at 40 nm distance from the channel pore (Additional file 2: Figure S2) (model parameters as described previously) [27]. This is important in validating the potential for Cav3 channels to provide the calcium necessary to promote CaM dissociation given that the coimmunoprecipitation between Cav3.1 and CaM was lost at calcium concentrations of 5 μM or more (Fig. 1e, f). Using this protocol we used the degree to which 50 mM [K]o evoked a Cav3-mediated calcium influx that could promote a loss of FRET between GFP-Cav3.1 and mKate-CaM.

**Fig. 3:**
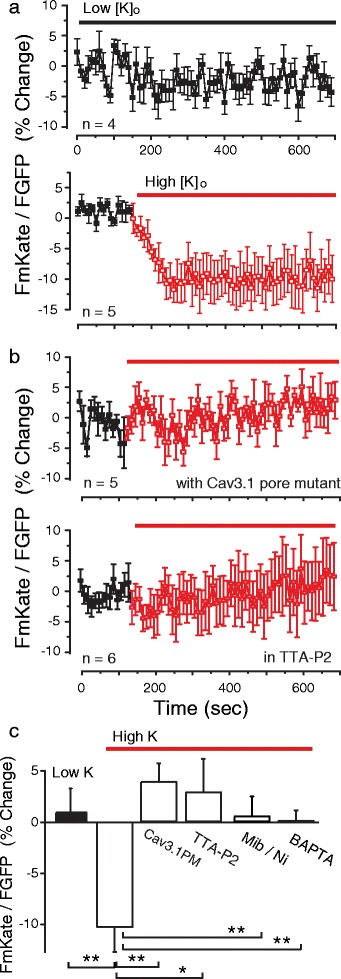
The Cav3-CaM association is lost upon Cav3 channel-mediated calcium influx. **a**, **b** Plots of FRET over time in tsA-201 cells coexpressing GFP-Cav3.1, mKate-CaM, and Kir2.1. Cells were exposed to *Low [K]o* (1 mM) or *High [K]o* (50 mM). **a** The FRET signal is stable in low [K]o for 11 min (*upper plot*) but is lost within 50 s upon perfusion of high [K]o (*lower plot*). **b** The decrease in FRET signal in high [K]o is blocked by expressing a Cav3.1 pore mutant that does not conduct calcium (*upper plot*), or 20 μM TTA-P2 (*lower plot*). **c** Bar plots of mean measures of FRET in the indicated conditions derived from 3 to 5 plates with 8–25 ROIs at time point 140 s. Mibefradil (Mib) 1 μM, Ni^2+^ 300 μM, BAPTA-AM 100 μM. Average values are mean ± SEM. * *p* < 0.05, ** *p* < 0.01, ns, not significant

Controls established that under conditions of 1.0 mM [K]o the mean level of FRET was stable over an initial 11 min period (−0.86 ± 2.63%, *n* = 11 ROIs) (Fig. 3a). Exposure to high [K]o then decreased FRET within 100 s (−9.1 ± 2.12%, *n* = 25 ROIs, *p* = 0.005) (Fig. 3a). To more carefully define the ability of Cav3.1-mediated calcium influx to account for the loss of FRET we substituted Cav3.1 cDNA with a GFP-Cav3.1 pore mutant that fails to conduct calcium (Fig. 3b: Additional file 3: Figure S3), and found that high [K]o exposure failed to reduce FRET under these conditions (4.55 ± 1.55%, *n* = 8 ROIs, *p* = 0.24) (Fig. 3b, c). Similarly, the loss of FRET promoted by high [K]o was blocked in the presence of the Cav3 calcium channel blockers TTA-P2 (20 μM) (Fig. 3b, c) (3.20 ± 4.86%, *n* = 8 ROIs, *p* = 0.58) or a combination of mibefradil (1 μM) and Ni^2+^ (300 μM) (−1.4 ± 1.87%, *n* = 25 ROIs, *p* = 0.99), or by 100 μM BAPTA-AM (1.2 ± 0.77%, *n* = 21 ROIs, *p* = 0.99) (Fig. 3c) (Additional file 4: Figure S4a, b). Attempts to repeat FRET measurements in dissociated hippocampal cultures were unfortunately not successful given a very low transfection efficiency of GFP-Cav3.1 and mKate-CaM. This resulted in an expression density that was too low to permit GFP excitation at 457 nm, as required to elicit mKate fluorescence as the acceptor of the GFP-mKate FRET pair. Nevertheless, the results of FRET obtained in tsA-201 cells provide strong evidence that a membrane depolarization that triggers calcium influx through Cav3.1 calcium channels is sufficient to induce a loss of FRET between GFP-Cav3.1 and mKate-CaM. The data are thus consistent with the results of coimmunoprecipitations as they indicate a constitutive association between Cav3.1 and CaM in physiologically low and nominally zero calcium conditions (BAPTA-AM), and a separation of the donor and acceptor proteins when the internal calcium concentration increased.

### Cav3.1 channel-mediated calcium influx activates αCaMKII

CaM is known to activate CaMKII to act as a second messenger involved in a wide range of functions that include gene transcription to synaptic plasticity [15, 16, 19]. Given indications that calcium influx through Cav3 channels reduced the association between Cav3.1-CaM detected at rest, we explored the potential for CaM to activate CaMKII in response to a depolarizing stimulus.

**Fig. 4:**
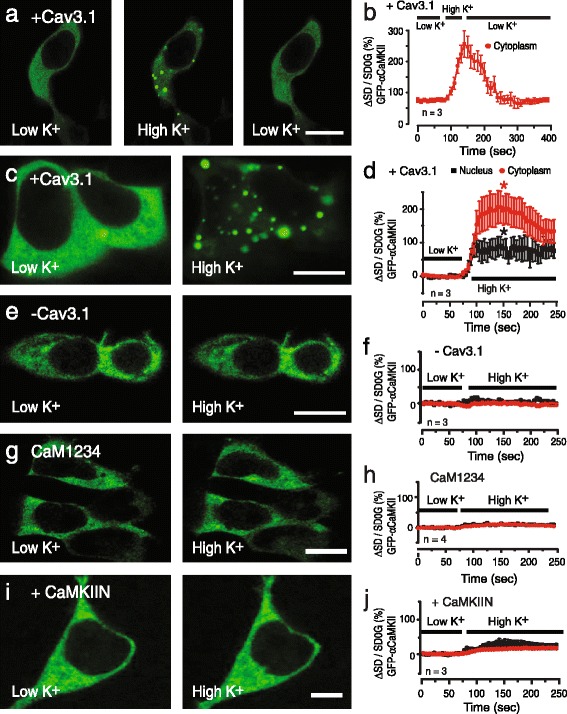
Cav3 channel-mediated calcium influx leads to αCaMKII aggregation. tsA-201 cells are cotransfected with GFP-αCaMKII, CaM, Cav3.1, and Kir2.1 and exposed to low [K]o (1.0 mM) or high [K]o (50 mM). Plots of the mean pixel variance of GFP-αCaMKII fluorescence in ROIs in the cytoplasm and nuclear regions shown at *right*. **a**, **b** In cells coexpressing Cav3.1 GFP-αCaMKII exhibits a diffuse cytoplasmic distribution in low [K]o that changes to aggregates in high [K]o, a pattern that is fully reversible upon returning to low [K]o. **c**, **d** Magnified images of the distribution of GFP-αCaMKII in low [K]o and after perfusing high [K]o, with plots indicating a restriction of clusters primarily to cytoplasm and peri-nuclear regions. **e**-**i** Formation of GFP-αCaMKII aggregates induced by high [K]o is prevented in cells lacking Cav3.1 (**e**, **f**), and blocked by substitution of CaM with CaM1234 (**g**, **h**), or by coexpression of CaMKIIN (0.3 μg) as an inhibitor of αCaMKII phosphorylation (**i**, **j**). Values are mean ± SD derived from *n* = 3 plates with 12–29 ROIs. Scale bars 10 μm.* *p* < 0.05

Activation of GFP-αCaMKII can be detected as a change in subcellular distribution from a diffuse to an aggregated state [17, 24]. We cotransfected tsA-201 cells with Cav3.1 and/or GFP-αCaMKII to monitor the distribution of αCaMKII at rest and following depolarization-activated Cav3-mediated calcium influx. CaM was coexpressed in all cells along with Kir2.1 to maintain a hyperpolarized resting potential. The distribution of GFP-αCaMKII was quantified by measuring the change in pixel variance of GFP fluorescence detected in cytoplasmic and nuclear compartments using defined ROIs (see Methods). Cells expressing GFP-αCaMKII and Cav3.1 exhibited a predominantly uniform cytoplasmic distribution in low (1.0 mM) [K]o (Fig. 4a, c). Perfusing high (50 mM) [K]o caused the GFP-αCaMKII label to rapidly form aggregates in the cytoplasm within 50 s (*p* < 0.05 measured at 150 s, *n* = 3) (Fig. 4a, c). These results are considered physiological given that the diffuse pattern of GFP-αCaMKII distribution was stable in low (1.0 mM) [K]o (Additional file 5: Figure S5a, b) and the GFP-αCaMKII aggregates formed in high [K]o fully reversed to a diffuse distribution within 1 min upon returning to low [K]o (Fig. 4a, b), as earlier reported [17].

Our ability to visualize GFP-αCaMKII aggregate formation during live cell imaging allowed us to test calcium-dependent events that lead to αCaMKII activation. One potential source for voltage-gated calcium entry that triggers αCaMKII activation is that of Cav1.x L-type calcium channels [15]. Although HVA calcium channels were not expressed in these experiments we conducted several controls to ensure that calcium influx was restricted to Cav3.1 channels in tsA-201 cells. First, cells expressing GFP-αCaMKII without Cav3.1 coexpression did not exhibit GFP-αCaMKII aggregation (Fig. 4e, f). Second, expressing a Cav3.1 pore mutant together with GFP-αCaMKII and CaM in tsA-201 cells fully blocked the high [K]o-evoked GFP-αCaMKII aggregation (Additional file 5: Figure S5c, d), despite the ability for the Cav3.1 pore mutant to conduct calcium (Additional file 3: Figure S3). We also confirmed that calcium influx was restricted to Cav3.1 channels by blocking L-type calcium channels with 30 μM Cd^2+^ (Additional files 5 and 6: Figures S5e-h and S6), and by finding that the high [K]o-induced aggregation of GFP-αCaMKII was blocked by 1 μM mibefradil and 300 μM Ni^2+^ (Additional file 5: Figure S5g, h). High [K]o-evoked GFP-αCaMKII aggregation was further blocked by 0.1 mM BAPTA-AM (Additional file 5: Figure S5i, j), indicating a requirement for an increase in [Ca]i subsequent to Cav3.1 channel activation. Together these data strongly suggest that all voltage-gated calcium influx that lead to αCaMKII activation in these experiments was conducted by Cav3.1 channels.

The dependence of GFP-αCaMKII aggregation upon calcium interactions with CaM was established by a loss of aggregate formation in high [K]o when CaM expression was substituted with the EF hand mutant CaM1234 (Fig. 4g, h). The activation of GFP-αCaMKII is known to require Ca^2+^/CaM to bind to the autoregulatory domain of αCaMKII to promote autophosphorylation and self-association of the αCaMKII holoenzymes into aggregates [17, 18]. αCaMKII activity and aggregation can also be inhibited through expression of CaMKIIN, a peptide that binds to the catalytic pocket of αCaMKII [24, 34]. To test if the depolarization-induced formation of GFP-αCaMKII aggregates depended on αCaMKII phosphorylation, we expressed GFP-αCaMKII and CaMKIIN in tsA-201 cells coexpressing Cav3.1 and CaM. These tests revealed that CaMKIIN blocked formation of GFP-αCaMKII aggregates following exposure to high [K]o (Fig. 4i, j). The outcome of these tests establish that a membrane depolarization that triggers Cav3 channel-mediated calcium influx activates αCaMKII through a calcium-dependent interaction with CaM and activation of αCaMKII.

### Cav3.1 calcium activation of αCaMKII depends on an intact Cav3.1 C-terminus

**Fig. 5:**
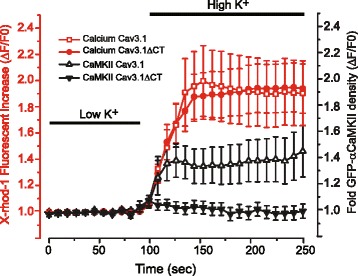
Cav3.1-mediated activation of αCaMKII depends on an intact Cav3.1 C-terminus. tsA-201 cells are transfected with Cav3.1 or a Cav3.1 mutant lacking the C-terminus (Cav3.1ΔCT) to remove a site for preassociation of CaM with the Cav3.1 channel and preloaded with 2 μM X-rhod-1 to detect changes in [Ca]i. Cells are maintained at rest in low [K]o (1 mM) or exposed to 10 min of high [K]o (50 mM), with all medium containing 30 μM Cd^2+^ to block HVA calcium channels. The extent and timecourse of a shift in GFP-αCaMKII distribution from diffuse to aggregates in the cytoplasm is compared to that of a change in fluorescent intensity of X-rhod-1 relative to the mean of control baseline (ΔF/F0). High [K]o increased [Ca]i to an equivalent relative level in cells expressing either Cav3.1 or Cav3.1ΔCT but GFP-αCaMKII aggregation is blocked in cells expressing the Cav3.1ΔCT mutant. The distribution of GFP-αCaMKII is measured as density for at least 25–34 ROIs from 4 to 5 plates. Values are mean ± SEM derived from *n* = 4–5 plates with 25–34 ROIs

It is important to define if the CaM that activates αCaMKII reflects CaM dissociated from the Cav3.1 channel or from a presumed cytoplasmic source of CaM subsequent to an increase in internal calcium concentration. These two possibilities were differentiated by simultaneously imaging the effects of high [K]o exposure on GFP-αCaMKII aggregation to that of changes in [Ca]i signaled by X-rhod-1 for cells expressing either Cav3.1 or a Cav3.1ΔCT construct that lacked the C-terminus and thus the CaM binding site (cf. Fig. 1c). Importantly, the Cav3.1ΔCT construct was confirmed to form functional T-type calcium channels (Additional file 7: Figure S7) that supported accumulation of intracellular calcium upon exposure to high [K]o (Fig. 5). Here we found that high [K]o-induced GFP-αCaMKII aggregation was blocked in cells expressing Cav3.1ΔCT but not Cav3.1, despite an equivalent increase in calcium fluorescence of X-rhod-1 (Fig. 5). These results are important in establishing that αCaMKII activation is mediated by Cav3.1-dependent calcium entry that requires the presence of a channel region capable of associating with CaM.

### Cav3 calcium influx triggers αCaMKII phosphorylation in neurons

**Fig. 6:**
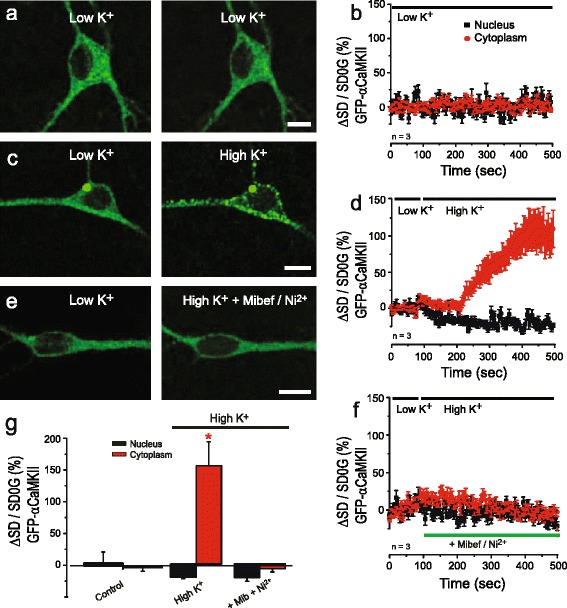
GFP-αCaMKII clustering in transfected neurons. Neurons are cotransfected with GFP-αCaMKII and Cav3.1 (unlabeled) and exposed to low [K]o (1.0 mM) or high [K]o (50 mM). **a**-**d** A diffuse distribution of αCaMKII-GFP is stable in low [K]o over time (**a**, **b**), with high [K]o promoting aggregation of GFP-αCaMKII (**c**, **d**). **e**, **f** Formation of GFP-αCaMKII aggregates induced by high [K]o is blocked in the presence of mibefradil (1 μM) and Ni^2+^ (300 μM). **g** Bar plots of the mean pixel variance of GFP-αCaMKII fluorescence for the tests in (**a**-**f**). Values are mean ± SD derived from *n* = 3 plates with 5–10 ROIs. Scale bars 10 μm.* *p* < 0.05

To determine if the Cav3.1-CaM-αCaMKII signaling cascade could be detected in neuronal populations we transiently transfected GFP-αCaMKII and Cav3.1 into dissociated cultures of hippocampal neurons. This test revealed a similar diffuse cytoplasmic distribution of GFP-αCaMKII fluorescence as found for tsA-201 cells in low levels of [K]o (Fig. 6a, b). But upon perfusion of high [K]o (50 mM) there was a substantial increase in the number of GFP-αCaMKII aggregates in the cytoplasm within 1.5 min (Fig. 6c, d). The aggregation of GFP-αCaMKII in high [K]o was again blocked in the presence 1 μM mibefradil/300 μM Ni^2+^ (Fig. 6e, f).

**Fig. 7:**
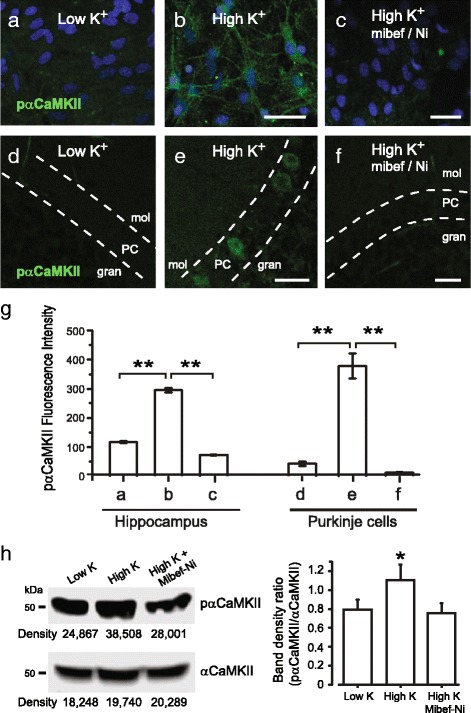
Cav3 calcium influx triggers αCaMKII phosphorylation in neurons. **a**-**f** Confocal images of cultured mouse hippocampal cells (**a**-**c**), and rat cerebellar tissue slices (**d**-**f**). Activated αCaMKII is identified by using a phospho-αCaMKII (pαCaMKII) antibody with additional DAPI label for nuclear staining in (**a-c**). Each preparation was maintained at rest in low [K]o (1 mM) or exposed to 10 min of high [K]o (50 mM) prior to fixation within 10 min of the end of high [K]o exposure. All results were derived from at least 3 separate experiments. In all neuronal cell tests calcium influx is restricted to Cav3 channels by maintaining cells in 30 μM Cd^2+^, 10 μM DNQX, 25 μM DL-AP5, and 1 μM TTX. **a**, **b** In hippocampal cell cultures high [K]o induces pαCaMKII labeling. **d**, **e** In cerebellar tissue slices high [K]o induces pαCaMKII labeling in Purkinje cell bodies. **c**, **f** High [K]o-induced labeling for pαCaMKII is blocked in both hippocampus (**c**) and cerebellum (**f**) in the presence of Cav3 channel blockers 1 μM mibefradil and 300 μM Ni^2+^. *Dashed lines* in (**d**-**f**) depict the boundaries of the Purkinje cell body layer. **g** Bar plots of the mean intensity of pαCaMKII fluorescence in hippocampal cell cultures and Purkinje cells in cerebellar tissue slices for panels (**a**-**f**) were quantified using ImageTrak software. Scale bars 10 μm. Values are mean ± SEM derived from *n* = 3–4 coverslips with 30–130 ROIs. **h** Western blot analysis to test the relative density of αCaMKII and p-αCaMKII labeling in cerebellar slice lysates in low or high [K]o, with mean bar plots of the band density ratio (pαCaMKII/αCaMKII) (Image J software). The high [K]o lysate has a higher band density than either low [K]o or high [K]o in the presence of mibefradil and Ni^2+^. Values are mean ± SEM derived from 3 separate experiments. * *p* < 0.05;** *p* < 0.01. PC, Purkinje cell layer; mol, molecular layer; gran, granule cell layer

The above results are important in indicating that Cav3.1-mediated calcium influx can mediate aggregation of GFP-αCaMKII in neurons, a process that tests in tsA-201 cells indicated should involve αCaMKII phosphorylation. To visualize the activation of phosphorylated αCaMKII we used immunolabel detection for pαCaMKII in both hippocampal cell cultures and cerebellar tissue slices exposed to 10 min of high [K]o (50 mM) medium in the presence or absence of mibefradil/Ni^2+^ (Fig. 7) (Additional file 8: Figure S8). Calcium influx was restricted to Cav3 calcium channels by conducting tests in medium containing 30 μM Cd^2+^, 10 μM DNQX, 25 μM or 100 μM DL-AP5, and 1 μM TTX, and tissues fixed for immunocytochemistry within 10 min after the high [K]o treatment. Under resting conditions of 1 mM [K]o hippocampal cell cultures and cerebellar tissue slices exhibited little if any detectable immunolabel for pαCaMKII (Fig. 7a–g). When exposed to high [K]o for 10 min hippocampal cells exhibited an increase in pαCaMKII immunolabel that highlighted cell somata and surrounding processes (Fig. 7b, g). Exposure of cerebellar tissue slices to high [K]o revealed an increase in pαCaMKII labeling in Purkinje cell bodies (Fig. 7e, g). The high [K]o-induced increase in pαCaMKII was again blocked in the presence of 1 μM mibefradil and 300 μM Ni^2+^ for both hippocampal cultures and cerebellar tissue slices (Fig. 7c, f, g). We also found a significantly reduced level of pαCaMKII immunolabel if hippocampal cultures were pre-exposed to a peptide inhibitor specific for pαCaMKII (AIP 10 μM; n 3–4, *p* < 0.01) (Additional file 8: Figure S8).

To complement the immunolabeling tests we further examined pαCaMKII protein density in cerebellar tissue slices exposed to low [K]o or high [K]o in the presence or absence of mibefradil/Ni^2+^-containing medium. Tissue was lysed in a medium containing the same final solution and prepared for Western blot analysis. In each case a band for pαCaMKII was detected, but the ratio of band densities (pαCaMKII/αCaMKII) was significantly greater for tissue exposed to high [K]o (*n* = 3; *p* < 0.05), a difference that was not present in tissues preexposed to mibefradil/Ni^2+^ (Fig. 7h).

## Discussion

CaM is known to establish an association with HVA calcium channels to provide feedback regulation of channel activity or downstream activation of CaMKII. The current study is important in showing that the Cav3.1 calcium channel exhibits a novel calcium-dependent interaction with CaM that also triggers CaM-dependent activation and phosphorylation of αCaMKII. Moreover, Cav3-mediated interactions can be detected in both tsA-201 cells as well as hippocampal cerebellar neurons.

### CaM association with Cav3.1 channels

The complexity and wealth of interactions that define CaM binding and modulation of HVA calcium channels have emerged over many years of work. An HVA calcium channel (e.g. Cav1.x) generally forms a pre-association with calcium free ApoCam at resting levels of internal calcium concentration, and a calcium-dependent increase in CaM binding affinity [13, 28–30]. The assays conducted here reveal that Cav3.1 channels exhibit a constitutive association with ApoCam at resting levels of calcium. The occurrence of FRET between Cav3.1 and CaM at resting levels of internal calcium confirms a close protein-protein interaction given that FRET requires two molecules to be positioned within 10–100 Å distance. CaM binding to HVA calcium channels involves at least an IQ domain on the calcium channel C-terminus, as well as an NSCaTE domain on the N-terminus [13, 35–40]. The amino acid sequence of the Cav3.1 C-terminus does not appear to present a clear IQ domain as defined for HVA channels, nor does it exhibit a canonical CaM binding motif. Our work establishes that the Cav3.1 C-terminus is required to detect a coimmunoprecipitation between CaM and Cav3.1, with GST pull down assays supporting a direct interaction between CaM and the C-terminal region. The precise amino acid residues on the C-terminus responsible for the Cav3.1-CaM interaction remain to be determined.

The Cav3.1-CaM association is further distinguished from CaM-HVA calcium channel interactions in that membrane depolarization promotes a loss of FRET between Cav3.1 and CaM. We note that a loss of FRET could reflect a repositioning of CaM on the Cav3.1 channel, however, the loss of coIP between Cav3.1 and CaM at [Ca] greater than 1 μM indicates a physical dissociation. Moreover, Cav3.1 calcium influx proves to be sufficient to promote Cav3.1-CaM dissociation, as established by preventing the depolarization-induced loss of FRET using Cav3 calcium channel blockers or by expressing a non calcium-conducting Cav3.1 channel pore mutant. These findings are important in revealing a dynamic interaction between Cav3.1 and CaM that is essentially opposite to that of HVA calcium channels.

### αCaMKII activation

Calcium influx can activate multiple forms of CaMKII that translocate to perinuclear compartments [15, 18, 41–47], with the current work addressing αCaMKII. Activation of αCaMKII has been traced to calcium influx via specific HVA calcium channels in different cell types [15, 17, 18, 24, 44]. The current study is the first to reveal an interaction between Cav3.1 calcium influx and subsequent activation of αCaMKII. An important question is the extent to which αCaMKII is activated specifically by CaM molecules that are preassociated with Cav3.1 under resting conditions and dissociate upon channel opening, as opposed to CaM activated in the cytoplasm subsequent to a Cav3.1-mediated increase in [Ca]i. A role for CaM that dissociates from the Cav3.1 channel was supported by our finding that expression of a Cav3.1 mutant lacking the C-terminus (removing the CaM binding site) blocked depolarization-induced GFP-αCaMKII aggregation despite an equivalent elevation of [Ca]i as found with intact Cav3.1 channels (Fig. 5). Others have shown that pH can augment calcium-dependent clustering of GFP-αCaMKII [17] so it is possible that this could contribute to our effects. But the current study establishes that calcium influx through the Cav3 channel class can initiate the steps that lead to an aggregation of GFP-αCaMKII that is fully reversible.

In neurons, synaptic stimulation induces long term changes in synaptic efficacy that involves CaMKII activation and translocation to perinuclear or synaptic regions [17, 46, 48, 49]. In this regard we found that exposure to high [K]o invoked a substantial increase in the number of GFP-αCaMKII aggregates in the cytoplasm of hippocampal neuronal cultures, and of pαCaMKII labeling in processes of cultured hippocampal neurons and in cerebellar Purkinje cell bodies. Moreover, all these effects were blocked by exposure to Cav3 channel blockers. These data verify that Cav3 channel-triggered activation of αCaMKII occurs in neuronal circuits known to exhibit long-term plasticity of synaptic function, including Cav3-mediated forms of LTP in cerebellar neurons [50, 51].

Thus, the sequence of events identified for Cav3.1-CaM-αCaMKII interactions differs from that of HVA calcium channels, revealing separate pathways to activate αCaMKII. A Cav3-initiated process is also distinct in that Cav3.1 channels can be activated over a larger voltage range than HVA calcium channels. Cav3.1-mediated activation αCaMKII may then be prominent in certain cell types or brain regions where these channels regulate cell excitability at even subthreshold potentials. However, we note that αCaMKII activation by way of Cav3.1 or HVA channel calcium influx is not mutually exclusive. Rather, we interpret these results as evidence that Cav3 calcium influx is sufficient to activate αCaMKII through a novel interaction with CaM, providing a means to complement the role for HVA calcium channels in triggering αCaMKII activation.

## Additional files

Additional file 1: Figure S1. Cav3.1 channels exhibit a calcium-dependent association with CaM. **a**, **b**. Tests for coimmunoprecipitation between Cav3.1 channels and CaM from rat brain lysate (**a**) or homogenates of tsA-201 cells coexpresssing Cav3.1 and CaM (**b**) in the presence of the indicated buffered levels of calcium. Cav3.1 coimmunoprecipitates with CaM in 0 and 100 nM calcium but not at 50 μM or 1 mM calcium. All results were derived from at least 3 separate experiments. (PDF 828 kb)

Additional file 2: Figure S2. A model of Cav3.1 channel conductance and increase in [Ca]. **a**. Plots of the voltage-dependence for activation (*blue line*) and inactivation (*orange line*) derived from steady-state voltage commands from whole-cell recordings of Cav3 current in Purkinje cells (modified from Engber et al. [27]. **b**. Calculated changes in internal calcium concentrations for a hemispherical compartment around the calcium source for a Cav3 channel with a single channel conductance of 9 pS. Voltage commands are applied from a holding potential of −75 mV in 10 mV steps to +10 mV and calcium internal concentration changes plotted for distances of 20 nm and 40 nm distance from a Cav3 channel, leading to a peak calcium concentration of 36 μM (20 nm) and 7 μM (40 nm) for a step to – 20 mV. (PDF 349 kb)

Additional file 3: Figure S3. Cav3.1 channel pore mutant constructs. A single amino acid mutation of Cav3.1 creates a pore mutant that does not conduct calcium current. Shown are representative recordings and the associated I-V plot of a transient low voltage-activated current measured in the Cav3.1 mutant expressed in tsA-201 cells. Superimposed recordings and plot illustrate the records obtained in normal bathing medium and following substitution of sodium in the bathing medium by 130 NMDG. (PDF 351 kb)

Additional file 4: Figure S4. Block of high [K]o-mediated loss of GFP-Cav3.1 - mKate-CaM FRET. Plots of the FRET signal over time in tsA-201 cells coexpressing GFP-Cav3.1 and mKate-CaM. Values are normalized to the mean value of all fluorescence measurements in the time period. **a**, **b**. Cells were exposed to Low [K]o (1 mM) or High [K]o (50 mM). A loss of FRET encountered upon exposure to high [K]o is prevented by the Cav3 channel blockers 1 μM mibefradil and 300 μM Ni^2+^ (**a**) and in the presence of 0.1 mM BAPTA-AM (**b**). Average values are mean ± SEM. (PDF 337 kb)

Additional file 5: Figure S5. Activation of αCaMKII depends on Cav3.1 calcium influx and an increase in [Ca]i. The distribution of GFP-αCaMKII tested in tsA-201 cells in low [K]o (1 mM) or high [K]o (50 mM) and indicated reagents. All cells are cotransfected with CaM and Kir2.1. Plots indicate mean pixel variance of fluorescence in ROIs in cytoplasm and nuclear regions. **a**, **b**. A diffuse distribution of αCaMKII-GFP is stable in low [K]o over time. **c**-**h**. The aggregation of αCaMKII-GFP depends on Cav3.1-mediated calcium influx, as shown when a Cav3.1 pore mutant (Cav3.1 PM) that does not conduct calcium is expressed (**c**, **d**), the ability for high [K]o to promote aggregation in the presence of 30 μM Cd^2+^(**e**, **f**) but not in the presence of mibefradil (1 μM) and Ni^2+^ (300 μM) (**g**, **h**). **i**, **j**. The dependence of αCaMKII-GFP aggregation induced by high [K]o exposure also depends on an increase in [Ca]i in being blocked by pre-expossure to BAPTA-AM (0.1 mM). Values are mean ± SD at 250 s derived from 3 to 4 plates with 14–19 ROIs. Scale bars 10 μm. (PDF 5411 kb)

Additional file 6: Figure S6. Selective block of Cav1 L-type calcium channels by external Cd^2+^. **a**, **b**. Shown are representative recordings of calcium current evoked in tsA-201 cells expressing either the Cav1.2 or Cav1.3 calcium channel isoforms (**a**) or Cav3.1 calcium channels (**b**), with associated mean I-V plots shown below. Perfusing 30 μM Cd^2+^ blocks both Cav1.2 and Cav1.3 channel isoforms (**a**) but not Cav3.1 (**b**). For Ca_V_1.2 channel expression included 2 μg each of human- α1C-PMT2, α2δ1-PMT2 and β1B-PMT2. For Ca_V_1.3 channel expression 2 μg each of human- α1D-GFP^37−^, α2δ1-pcDNA and β1B-pcDNA was used. Ca_V_1.2 and Ca_V_1.3 expressing cells were co-transfected with 100 ng eGFPN1 for identification of transfected fluorescence cells. Average values are mean ± SEM. (PDF 572 kb)

Additional file 7: Figure S7. Calcium conductance of Cav3.1 channel construct lacking the C-terminus. Representative recordings and I-V plot of T-type calcium current for a Cav3.1 channel construct lacking the C terminal region (Cav3.1ΔC), removing a key site for CaM association. Average values are mean ± SEM. (PDF 343 kb)

Additional file 8: Figure S8. Cav3-mediated activation of αCaMKII. Bar plots of the mean fluorescence intensity of pαCaMKII in cultured hippocampal cells in low [K]o (1 mM) and following exposure to 10 min of high [K]o (50 mM) prior to fixation within 10 min of the end of high [K]o exposure. Calcium influx is restricted to Cav3 channels by maintaining cells in 30 μM Cd^2+^, 10 μM DNQX, 100 μM DL-AP5, and 1 μM TTX. Labeling for pαCaMKII in the cytoplasm increases in high [K]o that is reduced by a peptide inhibitor specific for pαCaMKII (AIP 10 μM). Fluorescence intensity was quantified in ImageTrak software (see Methods). Values are mean ± SEM derived from *n* = 3–4 cover slips from at least 3 separate experiments with 29 ROIs. ** *p* < 0.01. (PDF 290 kb)

## References

[1] Simms BA, Zamponi GW (2014). Neuronal voltage-gated calcium channels: structure, function, and dysfunction. Neuron.

[2] Lambert RC, Bessaih T, Crunelli V, Leresche N (2014). The many faces of T-type calcium channels. Pflugers Arch.

[3] Cribbs L (2010). T-type calcium channel expression and function in the diseased heart. Channels (Austin).

[4] Mesirca P, Torrente AG, Mangoni ME (2015). Functional role of voltage gated Ca(2+) channels in heart automaticity. Front Physiol.

[5] Weiss N, Hameed S, Fernandez-Fernandez JM, Fablet K, Karmazinova M, Poillot C, Proft J, Chen L, Bidaud I, Monteil A (2012). A Ca(v)3.2/syntaxin-1A signaling complex controls T-type channel activity and low-threshold exocytosis. J Biol Chem.

[6] Jacus MO, Uebele VN, Renger JJ, Todorovic SM (2012). Presynaptic Cav3.2 channels regulate excitatory neurotransmission in nociceptive dorsal horn neurons. J Neurosci.

[7] Garcia-Caballero A, Gadotti VM, Stemkowski P, Weiss N, Souza IA, Hodgkinson V, Bladen C, Chen L, Hamid J, Pizzoccaro A (2014). The deubiquitinating enzyme USP5 modulates neuropathic and inflammatory pain by enhancing Cav3.2 channel activity. Neuron.

[8] Carabelli V, Marcantoni A, Comunanza V, de Luca A, Diaz J, Borges R, Carbone E (2007). Chronic hypoxia up-regulates alpha1H T-type channels and low-threshold catecholamine secretion in rat chromaffin cells. J Physiol.

[9] Coutelier M, Blesneac I, Monteil A, Monin ML, Ando K, Mundwiller E, Brusco A, Le Ber I, Anheim M, Castrioto A (2015). A recurrent mutation in CACNA1G alters Cav3.1 T-type Calcium-Channel conduction and causes autosomal-dominant cerebellar ataxia. Am J Hum Genet.

[10] Morino H, Matsuda Y, Muguruma K, Miyamoto R, Ohsawa R, Ohtake T, Otobe R, Watanabe M, Maruyama H, Hashimoto K (2015). A mutation in the low voltage-gated calcium channel CACNA1G alters the physiological properties of the channel, causing spinocerebellar ataxia. Mol Brain.

[11] Zamponi GW (2016). Targeting voltage-gated calcium channels in neurological and psychiatric diseases. Nat Rev Drug Discov.

[12] Ben-Johny M, Yue DT (2014). Calmodulin regulation (calmodulation) of voltage-gated calcium channels. J Gen Physiol.

[13] Pitt GS, Zuhlke RD, Hudmon A, Schulman H, Reuter H, Tsien RW (2001). Molecular basis of calmodulin tethering and Ca2+−dependent inactivation of L-type Ca2+ channels. J Biol Chem.

[14] Hudmon A, Schulman H, Kim J, Maltez JM, Tsien RW, Pitt GS (2005). CaMKII tethers to L-type Ca2+ channels, establishing a local and dedicated integrator of Ca2+ signals for facilitation. J Cell Biol.

[15] Wheeler DG, Barrett CF, Groth RD, Safa P, Tsien RW (2008). CaMKII locally encodes L-type channel activity to signal to nuclear CREB in excitation-transcription coupling. J Cell Biol.

[16] Lisman J, Yasuda R, Raghavachari S (2012). Mechanisms of CaMKII action in long-term potentiation. Nat Rev Neurosci.

[17] Hudmon A, Lebel E, Roy H, Sik A, Schulman H, Waxham MN, De Koninck P (2005). A mechanism for Ca2+/calmodulin-dependent protein kinase II clustering at synaptic and nonsynaptic sites based on self-association. J Neurosci.

[18] Hudmon A, Schulman H (2002). Neuronal CA2+/calmodulin-dependent protein kinase II: the role of structure and autoregulation in cellular function. Annu Rev Biochem.

[19] Catterall WA, Leal K, Nanou E (2013). Calcium channels and short-term synaptic plasticity. J Biol Chem.

[20] Lee JH, Daud AN, Cribbs LL, Lacerda AE, Pereverzev A, Klockner U, Schneider T, Perez-Reyes E (1999). Cloning and expression of a novel member of the low voltage-activated T-type calcium channel family. J Neurosci.

[21] Zamponi GW (2003). Calmodulin lobotomized: novel insights into calcium regulation of voltage-gated calcium channels. Neuron.

[22] McKay BE, McRory JE, Molineux ML, Hamid J, Snutch TP, Zamponi GW, Turner RW (2006). Ca(V)3 T-type calcium channel isoforms differentially distribute to somatic and dendritic compartments in rat central neurons. Eur J Neurosci.

[23] Lai MM, Hong JJ, Ruggiero AM, Burnett PE, Slepnev VI, De Camilli P, Snyder SH (1999). The calcineurin-dynamin 1 complex as a calcium sensor for synaptic vesicle endocytosis. J Biol Chem.

[24] Flynn R, Labrie-Dion E, Bernier N, Colicos MA, De Koninck P, Zamponi GW (2012). Activity-dependent subcellular cotrafficking of the small GTPase Rem2 and Ca2+/CaM-dependent protein kinase IIalpha. PLoS One.

[25] Khosravani H, Altier C, Simms B, Hamming KS, Snutch TP, Mezeyova J, McRory JE, Zamponi GW (2004). Gating effects of mutations in the Cav3.2 T-type calcium channel associated with childhood absence epilepsy. J Biol Chem.

[26] Christensen PC, Welch NC, Brideau C, Stys PK (2016). Functional ionotropic glutamate receptors on peripheral axons and myelin. Muscle Nerve.

[27] Engbers JD, Zamponi GW, Turner RW (2013). Modeling interactions between voltage-gated Ca (2+) channels and KCa1.1 channels. Channels (Austin).

[28] Ben Johny M, Yang PS, Bazzazi H, Yue DT (2013). Dynamic switching of calmodulin interactions underlies Ca2+ regulation of CaV1.3 channels. Nat Commun.

[29] Erickson MG, Alseikhan BA, Peterson BZ, Yue DT (2001). Preassociation of calmodulin with voltage-gated Ca(2+) channels revealed by FRET in single living cells. Neuron.

[30] Erickson MG, Liang H, Mori MX, Yue DT (2003). FRET two-hybrid mapping reveals function and location of L-type Ca2+ channel CaM preassociation. Neuron.

[31] Augustine GJ, Santamaria F, Tanaka K (2003). Local calcium signaling in neurons. Neuron.

[32] Choi J, Park JH, Kwon OY, Kim S, Chung JH, Lim DS, Kim KS, Rhim H, Han YS (2005). T-type calcium channel trigger p21ras signaling pathway to ERK in Cav3.1-expressed HEK293 cells. Brain Res.

[33] Lacinova L, Kurejova M, Klugbauer N, Hofmann F (2006). Gating of the expressed T-type Cav3.1 calcium channels is modulated by Ca2**+**. Acta Physiol (Oxf).

[34] Vest RS, Davies KD, O'Leary H, Port JD, Bayer KU (2007). Dual mechanism of a natural CaMKII inhibitor. Mol Biol Cell.

[35] Pate P, Mochca-Morales J, Wu Y, Zhang JZ, Rodney GG, Serysheva II, Williams BY, Anderson ME, Hamilton SL (2000). Determinants for calmodulin binding on voltage-dependent Ca2+ channels. J Biol Chem.

[36] Tang W, Halling DB, Black DJ, Pate P, Zhang JZ, Pedersen S, Altschuld RA, Hamilton SL (2003). Apocalmodulin and Ca2+ calmodulin-binding sites on the CaV1.2 channel. Biophys J.

[37] Dick IE, Tadross MR, Liang H, Tay LH, Yang W, Yue DT (2008). A modular switch for spatial Ca2+ selectivity in the calmodulin regulation of CaV channels. Nature.

[38] Asmara H, Minobe E, Saud ZA, Kameyama M (2010). Interactions of calmodulin with the multiple binding sites of Cav1.2 Ca2+ channels. J Pharmacol Sci.

[39] Kim J, Ghosh S, Nunziato DA, Pitt GS (2004). Identification of the components controlling inactivation of voltage-gated Ca2+ channels. Neuron.

[40] Simms BA, Souza IA, Zamponi GW (2014). A novel calmodulin site in the Cav1.2 N-terminus regulates calcium-dependent inactivation. Pflugers Arch.

[41] Cohen SM, Li B, Tsien RW, Ma H (2015). Evolutionary and functional perspectives on signaling from neuronal surface to nucleus. Biochem Biophys Res Commun.

[42] Ma H, Groth RD, Cohen SM, Emery JF, Li B, Hoedt E, Zhang G, Neubert TA, Tsien RW (2014). gammaCaMKII shuttles Ca(2)(+)/CaM to the nucleus to trigger CREB phosphorylation and gene expression. Cell.

[43] Deisseroth K, Heist EK, Tsien RW (1998). Translocation of calmodulin to the nucleus supports CREB phosphorylation in hippocampal neurons. Nature.

[44] Li B, Tadross MR, Tsien RW (2016). Sequential ionic and conformational signaling by calcium channels drives neuronal gene expression. Science.

[45] Wheeler DG, Groth RD, Ma H, Barrett CF, Owen SF, Safa P, Tsien RW (2012). Ca(V)1 and Ca(V)2 channels engage distinct modes of Ca(2+) signaling to control CREB-dependent gene expression. Cell.

[46] Deisseroth K, Bito H, Tsien RW (1996). Signaling from synapse to nucleus: postsynaptic CREB phosphorylation during multiple forms of hippocampal synaptic plasticity. Neuron.

[47] Bading H, Ginty DD, Greenberg ME (1993). Regulation of gene expression in hippocampal neurons by distinct calcium signaling pathways. Science.

[48] Marsden KC, Shemesh A, Bayer KU, Carroll RC (2010). Selective translocation of Ca2+/calmodulin protein kinase IIalpha (CaMKIIalpha) to inhibitory synapses. Proc Natl Acad Sci U S A.

[49] Fink CC, Meyer T (2002). Molecular mechanisms of CaMKII activation in neuronal plasticity. Curr Opin Neurobiol.

[50] Pugh JR, Raman IM (2008). Mechanisms of potentiation of mossy fiber EPSCs in the cerebellar nuclei by coincident synaptic excitation and inhibition. J Neurosci.

[51] Ly R, Bouvier G, Schonewille M, Arabo A, Rondi-Reig L, Lena C, Casado M, De Zeeuw CI, Feltz A (2013). T-type channel blockade impairs long-term potentiation at the parallel fiber-Purkinje cell synapse and cerebellar learning. Proc Natl Acad Sci U S A.

