# Supplementary figures and images for: A T-type channel-calmodulin complex triggers αCaMKII activation
Source: Mol Brain. 2017 Aug 11;10:37. doi: 10.1186/s13041-017-0317-8 (PMC5553682; doi:10.1186/s13041-017-0317-8)

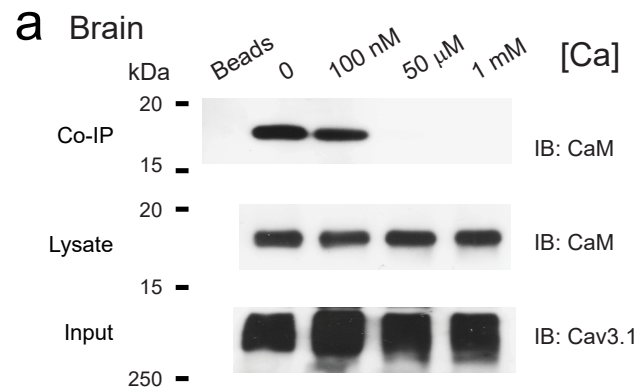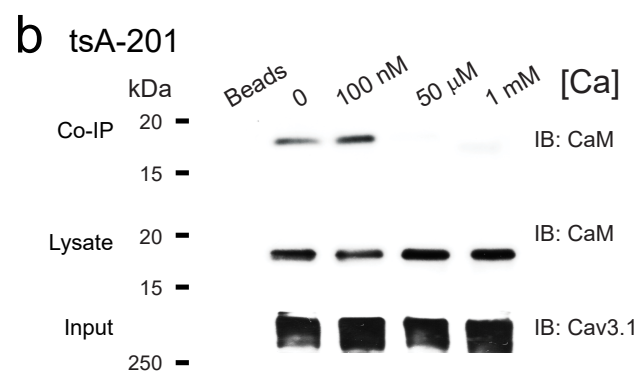

Supplement: Supplementary file 1 — Cav3.1 channels exhibit a calcium-dependent association with CaM. a, b. Tests for coimmunoprecipitation between Cav3.1 channels and CaM from rat brain lysate (a) or homogenates of tsA-201 cells coexpresssing Cav3.1 and CaM (b) in the presence of the indicated buffered levels of calcium. Cav3.1 coimmunoprecipitates with CaM in 0 and 100 nM calcium but not at 50 μM or 1 mM calcium. All results were derived from at least 3 separate experiments. (PDF 828 kb) [file 13041_2017_317_MOESM1_ESM.pdf]

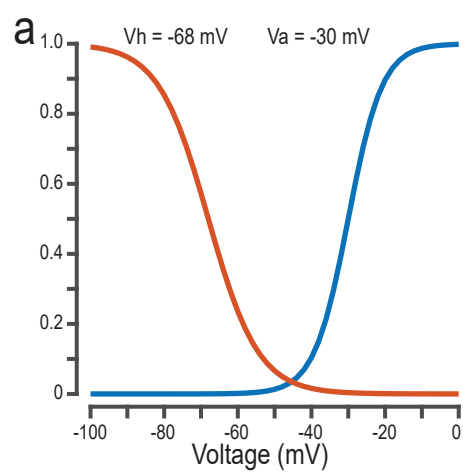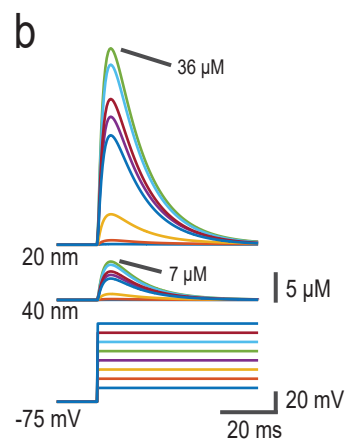

Supplement: Supplementary file 2 — A model of Cav3.1 channel conductance and increase in [Ca]. a. Plots of the voltage-dependence for activation (blue line) and inactivation (orange line) derived from steady-state voltage commands from whole-cell recordings of Cav3 current in Purkinje cells (modified from Engber et al. [27]. b. Calculated changes in internal calcium concentrations for a hemispherical compartment around the calcium source for a Cav3 channel with a single channel conductance of 9 pS. Voltage commands are applied from a holding potential of −75 mV in 10 mV steps to +10 mV and calcium internal concentration changes plotted for distances of 20 nm and 40 nm distance from a Cav3 channel, leading to a peak calcium concentration of 36 μM (20 nm) and 7 μM (40 nm) for a step to – 20 mV. (PDF 349 kb) [file 13041_2017_317_MOESM2_ESM.pdf]

# Cav3.1 pore mutant, 2 mM $\text{Ca}^{2+}$

— NaCl 130 mM, 2 mM  $\text{Ca}^{2+}$

— NMDG 130 mM, 2 mM  $\text{Ca}^{2+}$

$V_a = -36.3 \pm 1.12 \text{ mV (7)}$

$V_h = -63.3 \pm 1.12 \text{ mV (7)}$

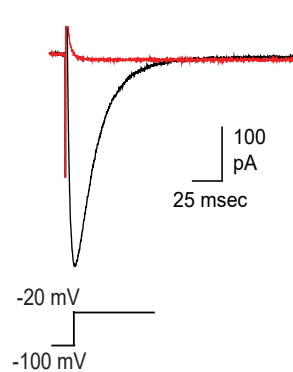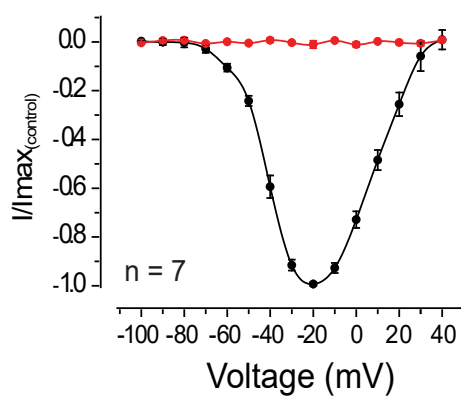

Supplement: Supplementary file 3 — Cav3.1 channel pore mutant constructs. A single amino acid mutation of Cav3.1 creates a pore mutant that does not conduct calcium current. Shown are representative recordings and the associated I-V plot of a transient low voltage-activated current measured in the Cav3.1 mutant expressed in tsA-201 cells. Superimposed recordings and plot illustrate the records obtained in normal bathing medium and following substitution of sodium in the bathing medium by 130 NMDG. (PDF 351 kb) [file 13041_2017_317_MOESM3_ESM.pdf]

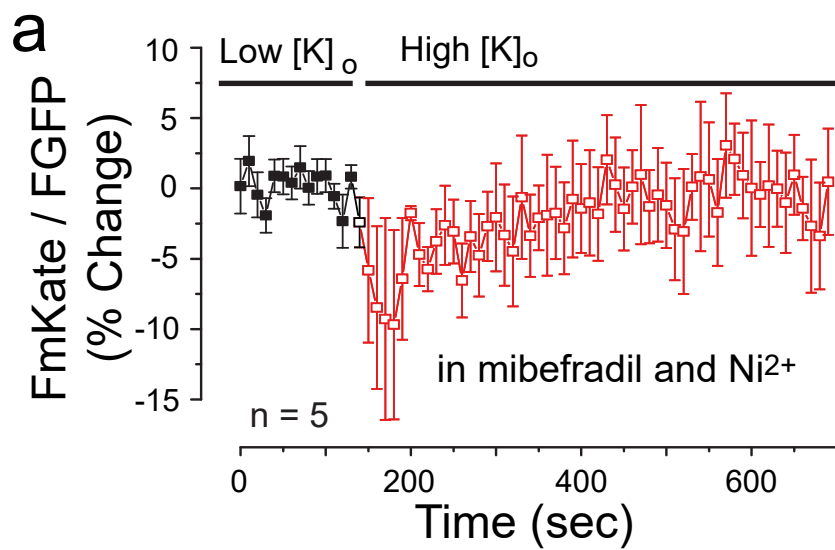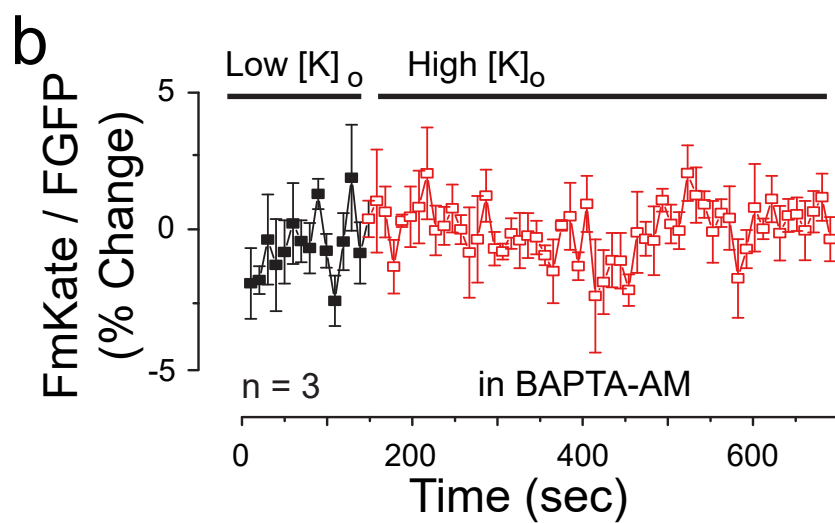

Supplement: Supplementary file 4 — Block of high [K]o-mediated loss of GFP-Cav3.1 - mKate-CaM FRET. Plots of the FRET signal over time in tsA-201 cells coexpressing GFP-Cav3.1 and mKate-CaM. Values are normalized to the mean value of all fluorescence measurements in the time period. a, b. Cells were exposed to Low [K]o (1 mM) or High [K]o (50 mM). A loss of FRET encountered upon exposure to high [K]o is prevented by the Cav3 channel blockers 1 μM mibefradil and 300 μM Ni2+ (a) and in the presence of 0.1 mM BAPTA-AM (b). Average values are mean ± SEM. (PDF 337 kb) [file 13041_2017_317_MOESM4_ESM.pdf]

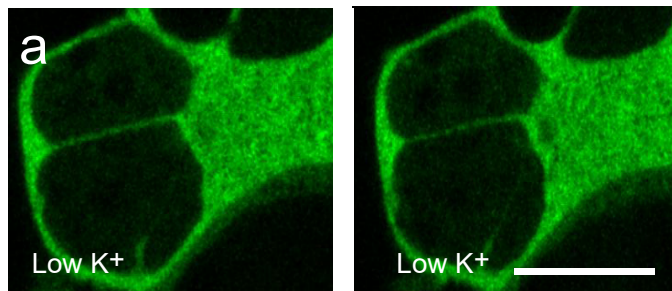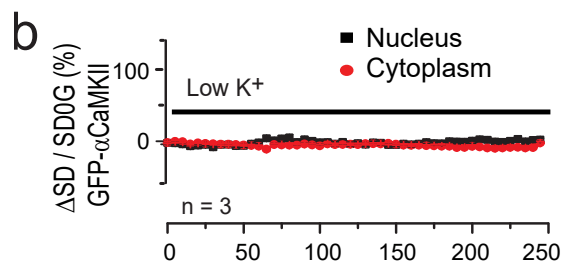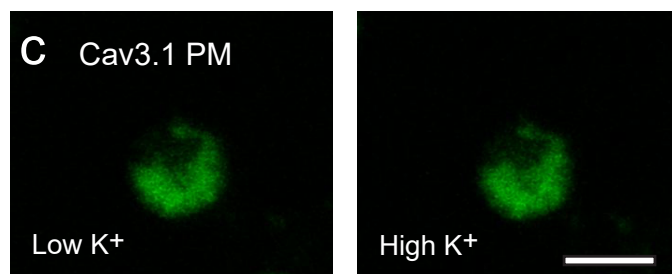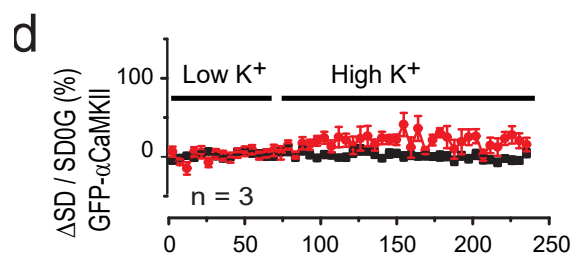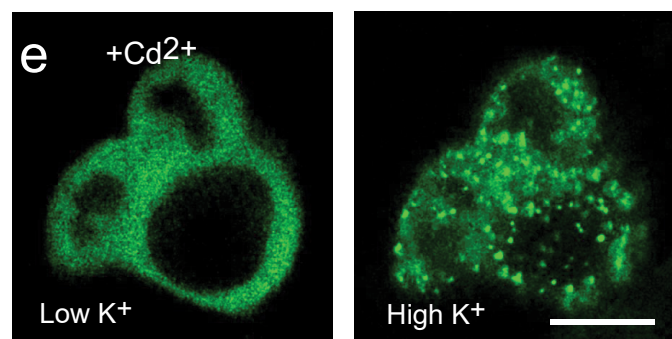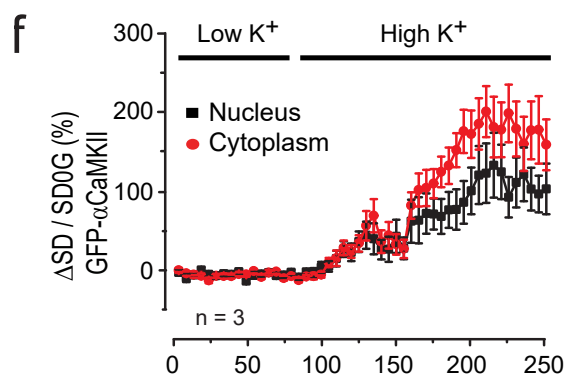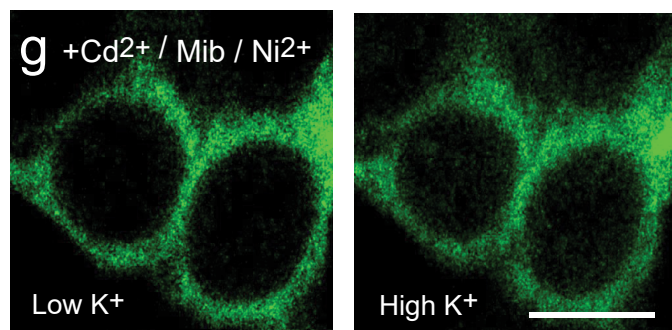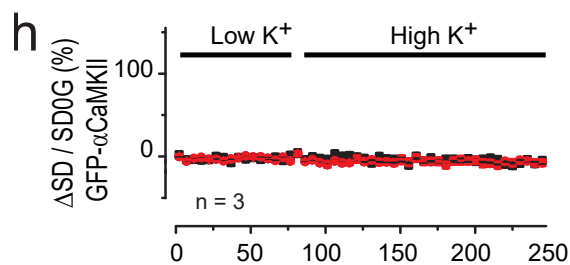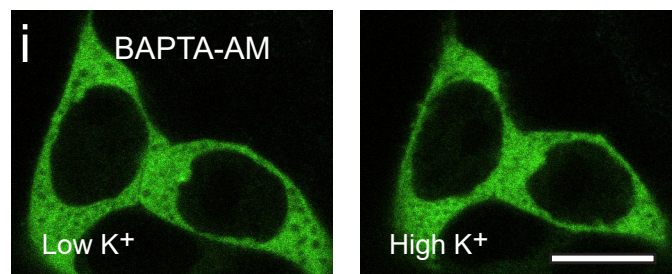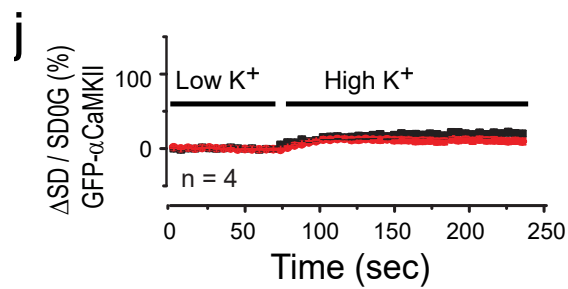

Supplement: Supplementary file 5 — Activation of αCaMKII depends on Cav3.1 calcium influx and an increase in [Ca]i. The distribution of GFP-αCaMKII tested in tsA-201 cells in low [K]o (1 mM) or high [K]o (50 mM) and indicated reagents. All cells are cotransfected with CaM and Kir2.1. Plots indicate mean pixel variance of fluorescence in ROIs in cytoplasm and nuclear regions. a, b. A diffuse distribution of αCaMKII-GFP is stable in low [K]o over time. c-h. The aggregation of αCaMKII-GFP depends on Cav3.1-mediated calcium influx, as shown when a Cav3.1 pore mutant (Cav3.1 PM) that does not conduct calcium is expressed (c, d), the ability for high [K]o to promote aggregation in the presence of 30 μM Cd2+(e, f) but not in the presence of mibefradil (1 μM) and Ni2+ (300 μM) (g, h). i, j. The dependence of αCaMKII-GFP aggregation induced by high [K]o exposure also depends on an increase in [Ca]i in being blocked by pre-expossure to BAPTA-AM (0.1 mM). Values are mean ± SD at 250 s derived from 3 to 4 plates with 14–19 ROIs. Scale bars 10 μm. (PDF 5411 kb) [file 13041_2017_317_MOESM5_ESM.pdf]

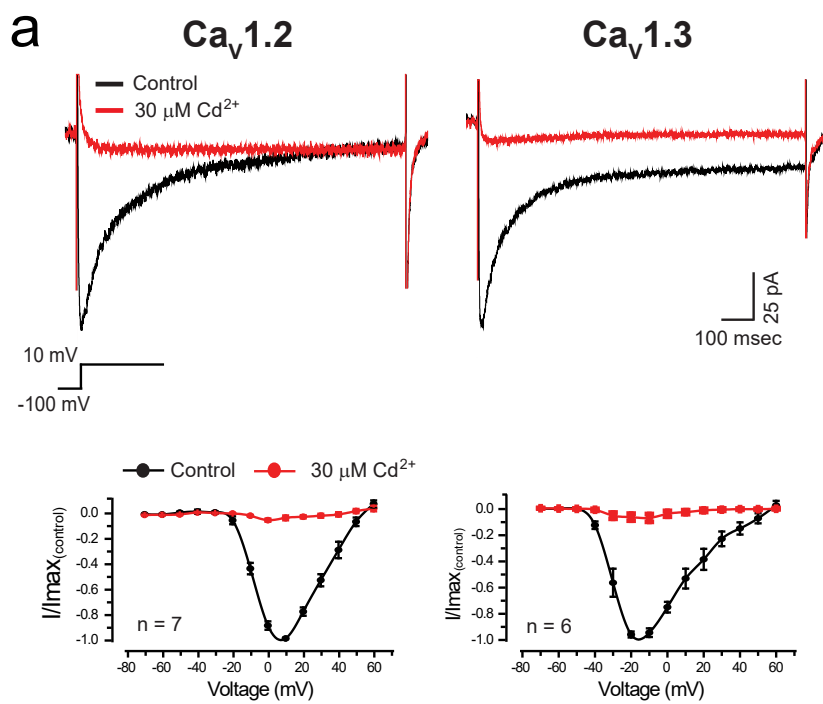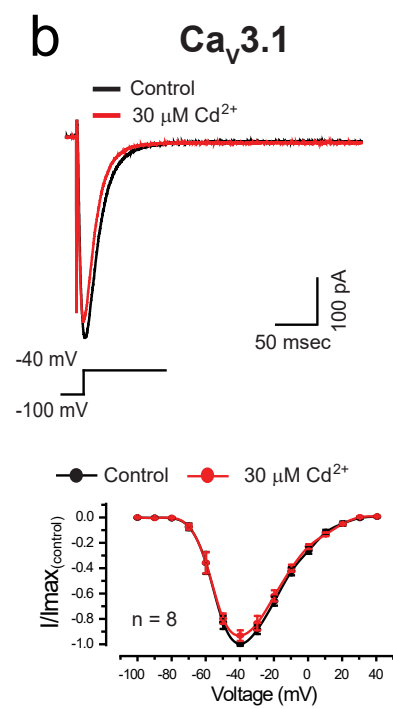

Supplement: Supplementary file 6 — Selective block of Cav1 L-type calcium channels by external Cd2+. a, b. Shown are representative recordings of calcium current evoked in tsA-201 cells expressing either the Cav1.2 or Cav1.3 calcium channel isoforms (a) or Cav3.1 calcium channels (b), with associated mean I-V plots shown below. Perfusing 30 μM Cd2+ blocks both Cav1.2 and Cav1.3 channel isoforms (a) but not Cav3.1 (b). For CaV1.2 channel expression included 2 μg each of human- α1C-PMT2, α2δ1-PMT2 and β1B-PMT2. For CaV1.3 channel expression 2 μg each of human- α1D-GFP37−, α2δ1-pcDNA and β1B-pcDNA was used. CaV1.2 and CaV1.3 expressing cells were co-transfected with 100 ng eGFPN1 for identification of transfected fluorescence cells. Average values are mean ± SEM. (PDF 572 kb) [file 13041_2017_317_MOESM6_ESM.pdf]

Cav3.1 $\Delta$ C, 2 mM Ca<sup>2+</sup>

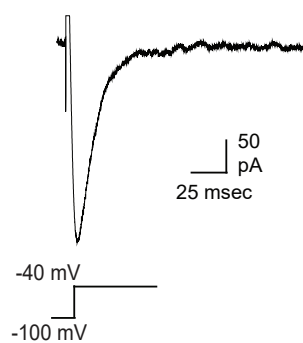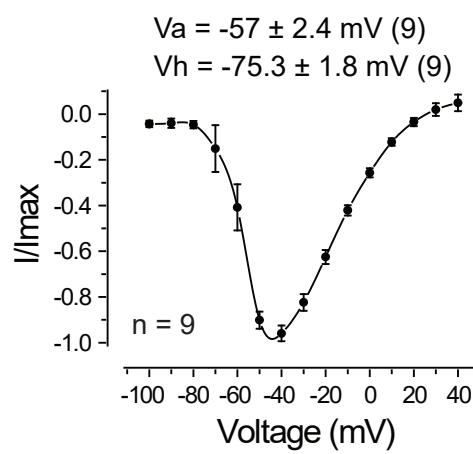

Supplement: Supplementary file 7 — Calcium conductance of Cav3.1 channel construct lacking the C-terminus. Representative recordings and I-V plot of T-type calcium current for a Cav3.1 channel construct lacking the C terminal region (Cav3.1ΔC), removing a key site for CaM association. Average values are mean ± SEM. (PDF 343 kb) [file 13041_2017_317_MOESM7_ESM.pdf]

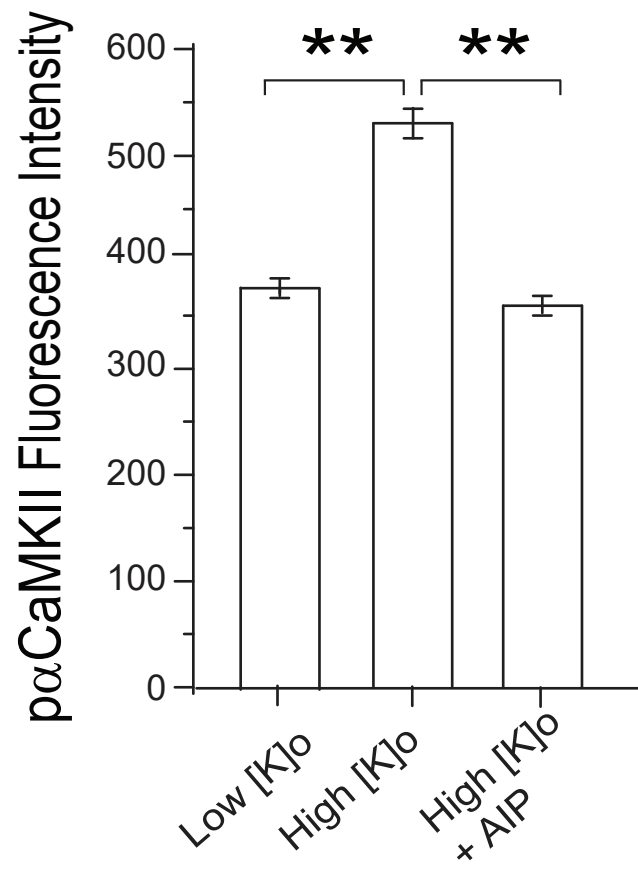

Supplement: Supplementary file 8 — Cav3-mediated activation of αCaMKII. Bar plots of the mean fluorescence intensity of pαCaMKII in cultured hippocampal cells in low [K]o (1 mM) and following exposure to 10 min of high [K]o (50 mM) prior to fixation within 10 min of the end of high [K]o exposure. Calcium influx is restricted to Cav3 channels by maintaining cells in 30 μM Cd2+, 10 μM DNQX, 100 μM DL-AP5, and 1 μM TTX. Labeling for pαCaMKII in the cytoplasm increases in high [K]o that is reduced by a peptide inhibitor specific for pαCaMKII (AIP 10 μM). Fluorescence intensity was quantified in ImageTrak software (see Methods). Values are mean ± SEM derived from n = 3–4 cover slips from at least 3 separate experiments with 29 ROIs. ** p < 0.01. (PDF 290 kb) [file 13041_2017_317_MOESM8_ESM.pdf]
